# Quantile regression for the statistical analysis of immunological data with many non-detects

**DOI:** 10.1186/1471-2172-13-37

**Published:** 2012-07-07

**Authors:** Paul HC Eilers, Esther Röder, Huub FJ Savelkoul, Roy Gerth van Wijk

**Affiliations:** 1 Department of Biostatistics, Erasmus MC-University Medical Center, PO Box 2040, 3000, CA, Rotterdam, The Netherlands; 2 Section of Allergology, Department of Internal Medicine (GK 324), Erasmus MC-University Medical Center, PO Box 2040, 3000, CA, Rotterdam, The Netherland; 3 Department of General Practice, Erasmus MC-University Medical Center, PO Box 2040, 3000, CA, Rotterdam, The Netherlands; 4 Cell Biology and Immunology Group, Wageningen University, PO Box 338, 6700, AH, Wageningen, The Netherlands

**Keywords:** Non-detects, Outliers, Robustness, Data analysis, Statistical, Quantile regression, Soluble biological markers, Immunological data

## Abstract

**Background:**

Immunological parameters are hard to measure. A well-known problem is the occurrence of values below the detection limit, the non-detects. Non-detects are a nuisance, because classical statistical analyses, like ANOVA and regression, cannot be applied. The more advanced statistical techniques currently available for the analysis of datasets with non-detects can only be used if a small percentage of the data are non-detects.

**Methods and results:**

Quantile regression, a generalization of percentiles to regression models, models the median or higher percentiles and tolerates very high numbers of non-detects. We present a non-technical introduction and illustrate it with an implementation to real data from a clinical trial. We show that by using quantile regression, groups can be compared and that meaningful linear trends can be computed, even if more than half of the data consists of non-detects.

**Conclusion:**

Quantile regression is a valuable addition to the statistical methods that can be used for the analysis of immunological datasets with non-detects.

## Background

Immunological parameters are hard to measure. A well-known problem [1] is the occurrence of values below the detection limit, the non-detects. In a project that we will use as an example in this paper, depending on the parameter, more than half of the data, concentrations of soluble biological markers in human blood, consists of non-detects.

Non-detects (NDs) are a nuisance in statistical analysis. An ad-hoc solution is to fill in values for the NDs, e.g. one half of the detection limit. This may be acceptable if only a few per cent of the observations are NDs. If there are many of them, estimated values of means, standard errors and trend lines will be unreliable and conclusions may be wrong.

NDs occur in many places in science and technology. They have received a lot of attention in the work of Helsel [2,3]. Although NDs are extremely common in immunology, the literature about them is not very extensive. An exception is the paper by Uh et al. [1] that studies a number of approaches to analyse datasets with NDs. In that paper quantile regression was not considered. We believe it to be a very useful tool, and like to share our experiences in this expository paper.

Most statistical methods develop a model for the expected values of the observations. In an analysis of variance (ANOVA) these will be the mean values for different groups. In the case of the regression line *y = ax + b,* the parameters *a* and *b* allow us to compute the expected value of an observation *y* for every x, which might be age or time or another covariate, that we are interested in. In addition, we can compute prediction intervals, in which a new observation will lie with a specified probability. This type of model belongs to the standard toolbox that most applied scientists learn these days in their statistics lessons. Modern statistical packages make it very easy to use them in practice.

Regression and ANOVA (which is a special case of regression), use the so-called principle of least squares: parameters like *a* and *b* in the example above, are computed in such a way that the sum of the squares of the residuals is minimized. The residuals are the differences between expected values, according to the model, and the observations. If a part of the observations is wrong, because of many NDs, the parameter estimates will be (very) wrong.

In this paper we propose to use quantile regression instead of the usual linear regression models. A simple example is provided by ANOVA. Instead of computing means per groups, one could compute the medians, also known as P50, the 50^th^ percentile. A familiar recipe for computing the median of a set of numbers is to sort them from low to high and pick the middle number in the sorted list. Half of the data will be below this number and the other half will be above it. The key point is that the actual values of the lowest observations play no role: what matters is that they are lower than the median. So if we would have 30% NDs and gave them small values, the computed median would still be the same.

If more than 50% of the observations are NDs, but less than 75%, we are still able to compute the P75, the number below which 75% of the data are found. In ANOVA we can still compare P75 in the different groups and look for interesting differences.

For a regression line, the sorting recipe will not work. However, in the last two decades a very useful generalization of regression modelling has become available, quantile regression. With this method we can estimate regression lines, which allow us to compute for *y* a percentile of our choice for any value of *x.* The only condition is that all NDs lie below the line. With many NDs, as in our example data set, this means that it is not possible to compute a line for the median, but that the P75 is sufficient.

The outline of the paper is as follows. First, we introduce quantile regression. We have tried to limit the amount of technical material, keeping in mind the expected statistical level of our audience. We also show in this section how the required computations can be done relatively easily with the R system and the package *quantreg*[4]. Then we apply quantile regression to a real data set, with an extremely high number of NDs. The paper ends with a short discussion.

## Methods

### Quantile regression

In this paper the words quantiles and percentiles will be used repeatedly. To avoid confusion we first give their precise meaning. The 90-th percentile is the number below which 90% of the data lie. It is also the 0.9 quantile. So, when we use percentages we talk of percentiles, and when we use fractions we talk of quantiles.

In the Introduction we described the familiar sorting algorithm for computing percentiles. It has a strong intuitive appeal, and it is easy to implement, or even to do by hand. However, it cannot be generalized to the case of a regression line or more complicated models. Fortunately there exists another, more flexible approach, based on optimisation.

The mean of n observations, y_1_ to y_n_, is computed as $\bar{y}=\sum_{i}y_{i}/n$. Averaging is such a familiar process that one usually does not give much thought to the fact that the sum of squares

(1) $$S_{2}=\sum_{i}{\left(y_{i}-\mu \right)}^{2}$$

is minimized when μ = $\bar{y}$, the mean of y. The sum of squares is stated explicitly in more complicated models like a linear regression line and it leads to explicit expressions for optimal values of the parameters in the model. This is an extremely powerful statistical tool.

For percentiles we can also introduce a function that has to be minimized, in such way that the desired percentile minimizes it. Compared to the sum of squares, two changes are needed:

1) replace the squares by absolute values, and 2) give different weights to positive and negative residuals. The residuals are the differences between the observations and the percentile that is being computed. As a formula: minimize

(2) $$S_{1}=\sum_{i}w_{i}(p)|y_{i}-q(p)|$$

Here *q*(*p*) is the *p*-quantile (the 100*p* percentile) for a chosen value *p* (with 0 < p < 1) and *w*_*i*_(*p*) is the weight of observation *i*, computed as

(3) $$w_{i}\left(p\right)=p\;\text{ if }\;y_{i}>q\left(p\right)$$

(4) $$w_{i}\left(p\right)=1-p\;\text{ if }\;y_{i}\leq q\left(p\right)$$

In the case of the 0.9-quantile, the positive residuals get a nine times larger weight (i.e. 0.9) than the negative ones (i.e. 0.1). It might not be directly obvious why this procedure leads to the desired quantile, but after some mathematical adjustments one finds indeed that 90% of the observations have to be below q_0.9_ to minimize *S*_1_. An intuitive explanation is that every observation above the quantile has to be balanced by nine below it.

Now that we have an optimisation criterion, it is very easy to extend the quantile idea to more complicated models. In the case of a linear regression line, the function to be minimized is

(5) $$S_{1}=\sum_{i}w_{i}(p)|y_{i}-a(p)-b(p)x_{i}|$$

It will be clear that we can generalize this to more complicated models. Notice that generally the values of *a*(*p*), the intercept, and *b*(*p*), the slope, change with *p.*

It is easy to state the function that has to be minimized, but computing the solution is harder than for classical models (based on least squares). Fortunately, there is excellent open-source software available, free of charge. We did our computations using the quantreg package for the statistical software system R [4]. Fitting a linear regression line for the 90th percentile is as simple as writing *model = rq(y ˜x, tau = 0.9).* The parameter *tau * corresponds to *p* in our formulas.

With quantile regression it is not possible to get *p*-values for model coefficients like slope and intercept; instead the *quantreg* package delivers 95% confidence intervals (which actually are more useful).

Although it is not an issue here, quantile regression is very robust against outliers, in contrast to the mean and least squares regression. Also a normal distribution of errors is not assumed.

For those interested in statistical backgrounds of quantile regression, we can recommend a paper by Koenker and Portnoy [5] and a book by Koenker [6]. An interesting paper from an applied point of view (i.e. that of ecologists) is the one by Cade and Noon [7].

## Results

### An implementation

To illustrate the use of quantile regression in immunology, we use data from the STARDROP-study, a randomized placebo-controlled trial in 204 youngsters (6–18 years) with hay fever. A detailed description can be found in Röder et al [8]. The main aim of the study was to determine the effect of sublingual immunotherapy (SLIT) with grass pollen allergen on nose and eye symptoms (e.g. sneezing and itchy eyes). Allergen-specific immunotherapy consists of the repeated administration of the allergen to which the patient is allergic, with the intention to modulate the response of the immune system to the allergen [9]. In the case of SLIT, the allergen is administered under the tongue by drops or tablets. In a sub-study, the effect of SLIT and other factors on the immune system was assessed by measuring the levels of soluble biological markers (SBMs) in plasma during the trial. Serum samples were collected at five time points during the two-year treatment period: baseline (T0), after 6 months (T1), after 12 months (T2), after 18 months (T3) and after 24 months (T4). All samples were collected outside the grass pollen season. The samples were analysed for their IL-12, IFN-γ, TNF-α, IL-10, IL-13, sICAM-1, sE-selectin and sIL-2Receptor content. The following factors were studied: treatment, age, gender, cohort (i.e. the year of inclusion), time points and co-sensitisation to birch pollen and house dust mite.

Out of the 203 youngsters included in this sub-study, 103 subjects were observed all 5 times and 74 only once. The 26 remaining subjects were observed 2 to 4 times.

We start by presenting histograms of the measurements, emphasizing the need for a statistical method that can handle a large proportion of NDs. Then we compute trends with age using quantile regression.

### Distributions and non-detects

The samples were analysed in two parts, because an interim-analysis had to be presented to our sponsor. As a consequence, two different assays with different detection limits were used.

Initially, for the time points T0, T1 and T2, the production of the SBMs was detected with Enzyme-Linked Immunosorbent Assay (ELISA). The sensitivity limits for quantitative determinations were 1.19 pg/ml (IFN-γ), 1.15 pg/ml (IL-10), 7.85 pg/ml (IL-12), 5.21 pg/ml (IL-13), 8.81 pg/ml (TNF-α), 13.40 pg/ml (sIL-2R), 0.11 ng/ml (sE-selectin), and 1.43 ng/ml (sICAM-1). For the later time points T3 and T4, the SBM production was measured with Cytometric Bead Assay Flex sets (CBA). The sensitivity limits for quantitative determinations were 0.3 pg/ml (IFN-γ), 2.3 pg/ml (IL-10), 2.2 pg/ml (IL-12), 1.6 pg/ml (IL-13), 0.7 pg/ml (TNF-α), 12.5 pg/ml (sIL-2R), 5 pg/ml (sE-selectin), and 0.23 ng/ml (sICAM-1). The measurements above the detection limits were not affected by the change in assays.

We chose to apply the detection limits of one method to all data. The detection limits of the first method (ELISA) were used because these limits were higher than those of the second method (CBA) for all SBMs except IL-10. For IL-10 the detection limits of both methods were used. All values below the detection limit were replaced with the value between 0 and the detection limit.

For the analysis and presentation of the data in this implementation we use the logarithms (to base 10) of concentrations. The highest concentrations measured were around 5000 pg/ml, the lowest were always at the detection limit. Because of the enormous range of the concentrations, the highest ones being more than a 1000 times higher than the lowest, we work exclusively on the logarithmic scale.

**Figure 1:**
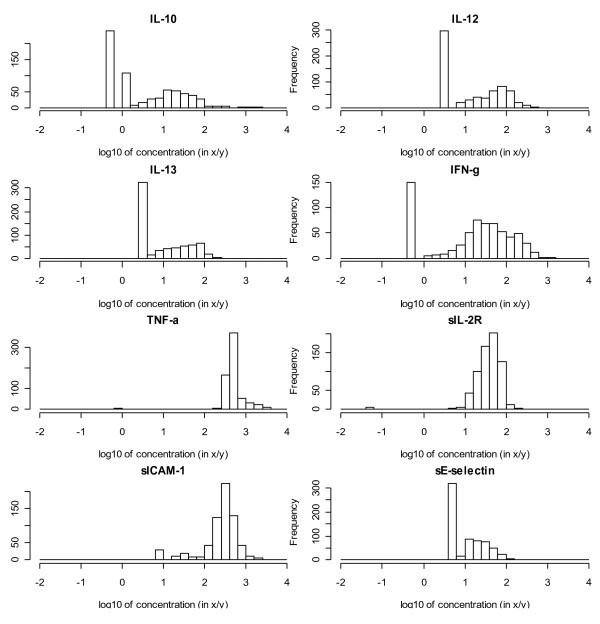
Histograms of (logarithms) of SBM concentrations, all five time points combined.

**Figure 2:**
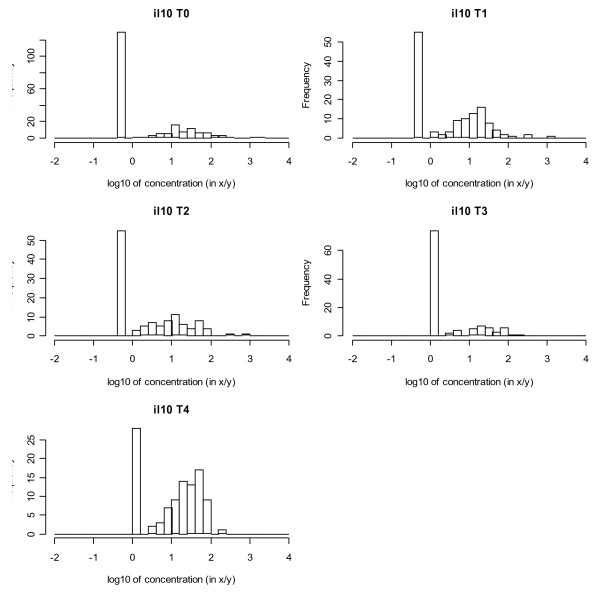
**Histograms of (logarithms) of IL-10 concentrations, for each of the five time points.** Legend: T0, baseline. T1-2-3-4, follow-up after 6-12-18-24 months respectively.

Figure 1 shows histograms of the logarithms (expressed as pg/ml or ng/ml; to base 10) of the concentrations of the eight SBMs. For IL-10, IL-12, IL13, IFN-γ, TNF-α and sIL-2R the percentage of NDs ranged from 4% up to 52%. The NDs clearly stick out as isolated bars at the left side of the histograms and are relatively close to the rest of the distribution. For sICAM-1 and sE-selectin they are small and at a large distance. For those SBMs the fraction of NDs was below 1%. In fact one could well apply a classical statistical analysis to these SBMs after discarding the few NDs. The number of NDs also varied between time points, as demonstrated for IL-10 in Figure 2. Also visible in this figure is the change in detection limit between T2 and T3. Except for IL-12 at one time point, the percentage of non-detects did not exceed 75%.

Summarizing, due to changes in detection limits and the presence of a substantial number of NDs for some SBMs, classical statistical analyses, like ANOVA and regression, could not be applied to this dataset.

### Trends and quantile regression

One of the research questions was to determine whether concentrations of SBMs change with age. We use quantile regression for P75, the 75^th^ percentile. In general any quantile level can be modelled and it would be more attractive to model P50, the median. The percentage of non-detects for some SBMs, however, was more than 50% and therefore we have to settle for a higher percentile. We chose P75, and we are aware that this arbitrary. Any percentile can be chosen, as long as it results in a quantile regression line that is above the values of non-detects everywhere.

**Figure 3:**
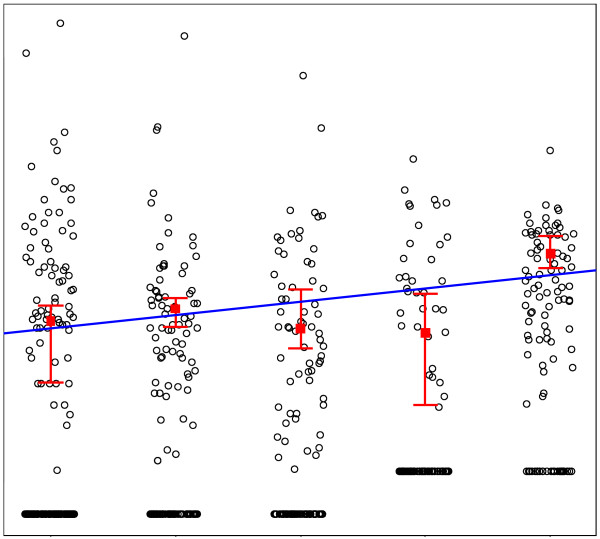
**The five observation time points of IL-10.** Legend: The data points have been "jittered" (a random horizontal shift between −0.2 and 0.2) for better visibility. For each time point the midpoint (coefficient) for the P75 with 95% confidence interval are shown. The intervals are not symmetric around the midpoint. The line represents the P75 quantile regression line for the logarithm of the concentration.T0, baseline. T1-2-3-4, follow-up after 6-12-18-24 months respectively.

**Figure 4:**
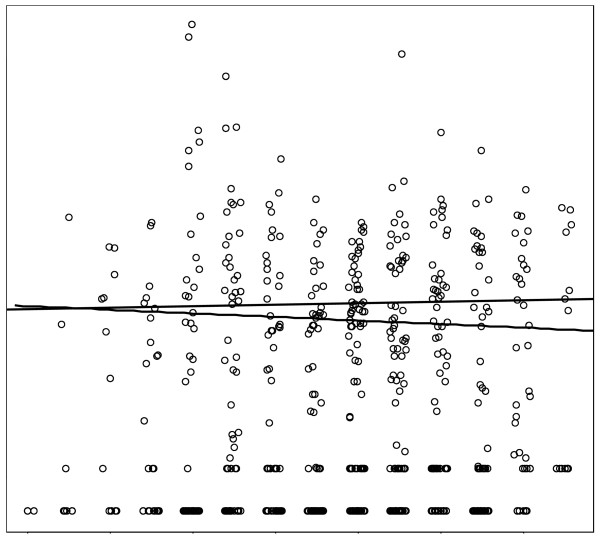
**Trend for the 75th percentile (P75) of IL-10 with age.** Legend: The data points have been "jittered" (a random horizontal shift between −0.2 and 0.2) for better visibility. The full line is obtained by quantile regression on age only, the broken line with the observation time point as an additional factor.

Before a quantile regression line on age only was calculated, the influence of the variable “time point” was explored. Figure 3 shows an example, again for IL-10. The data points have been “jittered” by adding small (between −0.2 and 0.2) random numbers to the time point, shifting the dots in horizontal direction and thereby giving an impression of the distribution of the concentrations. For each time point the P75 midpoint with its 95% CI is presented. The line represents the P75 quantile regression line with only “time point” as an explanatory variable. Because this line showed a slightly increasing trend, “time point” was incorporated as an additional factor (i.e. with a separate coefficient for each time point) in the analysis on the effect of age. Also visible in Figure 3 is the change in the analytical procedure between T2 and T3, leading to an increase in detection limits. As stated before, the measurements above the detection limits were not affected by the change in assays. Figure 4 shows the results of the analysis on the influence of age on the IL-10 levels. Age was rounded to integer years and again the data points have been “jittered” in the horizontal direction for better visibility. Two lines are presented in Figure 4. The full line is the result of quantile regression on age only. The broken line adds the factor “time point” as an explanatory variable. Thus, age appears not to have an effect on this SBM.

## Discussion

Immunological datasets often contain many non-detects. When a signal produced by the stimulant is too small for the instrumentation to discriminate the signal from the background noise, a value cannot be determined precisely. Values below a given detection limit are called non-detects (NDs). The presence of NDs will cause the data to be left-censored and special attention should be paid to selecting the appropriate statistical method to analyse such a censored dataset.

Several statistical methods are being used to deal with NDs. Uh et al. evaluated the performance of several commonly used methods in immunology and more advanced methods used in other fields such as environmetrics and econometrics via simulation studies [1]. Two often-used approaches, deletion or single value substitution followed by linear regression, did not perform well. Because NDs are not missing at random, bias can be expected when dropping NDs. Uh et al. showed that even with a ND proportion of 10%, the bias was unacceptable. Substitution of NDs with 0, half of the detection limit or the detection limit itself, followed by linear regression, underestimated the variance. Two more sophisticated methods, the TOBIT method and the multiple imputation technique, performed well but only when the proportion of NDs was less than 30% and 50%, respectively. In our dataset, for some markers the percentage of NDs was higher. Furthermore, use of the TOBIT method requires that the normality assumption is met. Like in our dataset, immunological measurements are often positively skewed and even after logarithmic transformation normality cannot always be achieved. Therefore, we had to seek for a method that could handle large proportions of NDs with no assumptions on the underlying distribution. We explored the use of quantile regression, a generalization of percentiles to regression models. Like for the computation of simple percentiles, the only information that is being used is whether observations are below or above estimated model values. If the number of NDs is not too large, one can estimate models for P50, the median. In extreme cases, like for some immunological markers in the data set we used as an example, it is necessary to go to higher percentiles. In fact we chose P75.

We illustrated quantile regression with data from a clinical trial in youngsters with hay fever, in which the effect of immunotherapy treatment and other factors on the immune system was evaluated by measuring levels of soluble biological markers (SBMs). We showed that groups can be compared and that meaningful linear trends can be computed, even with very large fractions of NDs. The slope of the regression line for a percentile is the same as that for the mean in the case of a linear relationship plus errors with a constant variance, the common default assumption in linear regression. That means that the estimated slope for the P75 is also a very good estimate for the usual regression slope that would be obtained if NDs did not occur.

We have not discussed efficiency. It is true that quantile regression uses less information, that is, only the signs of residuals, disregarding their size, leading to wider confidence intervals and consequently loss of power. This means that if data are complete (no NDs), estimated classical regression coefficients have more narrow confidence intervals than those obtained from quantile regression. But this knowledge does not help us much if we have many NDs. When analysing our data set, we chose one percentile level, 75% for all variables. In principle it could vary with the fraction of NDs, so that for some variables P50 could have been chosen. However, we felt that this would have made the interpretation more complicated.

The data have been analysed as 662 independent observations, which is a limitation, as 558 observations represent multiple observations on 129 participants. In the world of standard least squares statistical methods, one would use repeated measure ANOVA or a mixed model for a proper analysis. Unfortunately similar technology is not yet developed enough for quantile regression, although research is ongoing. NDs can, however, generate unpleasant complications when using mixed models. It might happen that all or most measurements of some of the subjects are NDs. Consequently mixed models, which rely on fitting (restricted) individual coefficients to subjects, might be difficult to use. As far as we know, no statistical technology is yet available to handle mixed models with NDs.

An alternative approach is reducing the problem to logistic regression, after setting proper thresholds (with a different value for each variable). Choosing the thresholds can be a matter of debate, which is avoided in quantile regression. In fact the quantile regression line acts as a “moving threshold” in such a way that on average (in the case of P75) a quarter of the data lies above it. Nevertheless, thresholding an logistic regression could be an interesting venue for longitudinal data modelling, because mixed model technology for binary responses is available.

In our application trends are so weak that there is no need for anything more complex than a straight line. But we remark that the *quantreg* package also allows computing more complex non-parametric trends.

## Conclusions

Quantile regression is a valuable addition to the statistical methods that can be used for the analysis of immunological datasets with non-detects.

## Abbreviations

ANOVA, Analysis of variance; CBA, Cytometric Bead Assay; ELISA, Enzyme-Linked Immunosorbent Assay; ND, Non-detect; SBM, Soluble biological marker; SLIT, Sublingual immunotherapy.

## Competing interests

The author(s) declare that they have no competing interests.

## Authors’ contributions

PE performed the statistical analysis. HS was responsible for the soluble biological marker measurements. PE and ER drafted the manuscript. All authors interpreted the data and critically reviewed, edited and approved the written manuscript.
